# Local Sleep Slow-Wave Activity Colocalizes With the Ictal Symptomatogenic Zone in a Patient With Reflex Epilepsy: A High-Density EEG Study

**DOI:** 10.3389/fnsys.2020.549309

**Published:** 2020-10-21

**Authors:** Eric W. Moffet, Ruben Verhagen, Benjamin Jones, Graham Findlay, Elsa Juan, Tom Bugnon, Armand Mensen, Mariel Kalkach Aparicio, Rama Maganti, Aaron F. Struck, Giulio Tononi, Melanie Boly

**Affiliations:** ^1^Department of Neurology, University of Wisconsin-Madison, Madison, WI, United States; ^2^Ken and Ruth Davee Department of Neurology, Northwestern University Feinberg School of Medicine, Chicago, IL, United States; ^3^Department of Psychiatry, University of Wisconsin-Madison, Madison, WI, United States; ^4^Department of Philosophy, Vrije Universiteit Amsterdam, Amsterdam, Netherlands; ^5^Department of Psychology, University of Amsterdam, Amsterdam, Netherlands

**Keywords:** high-density EEG, reflex epilepsy, sleep, slow-wave activity, delta power

## Abstract

**Background**: Slow-wave activity (SWA) during non-rapid eye movement (NREM) sleep reflects synaptic potentiation during preceding wakefulness. Epileptic activity may induce increases in state-dependent SWA in human brains, therefore, localization of SWA may prove useful in the presurgical workup of epileptic patients. We analyzed high-density electroencephalography (HDEEG) data across vigilance states from a reflex epilepsy patient with a clearly localizable ictal symptomatogenic zone to provide a proof-of-concept for the testability of this hypothesis.

**Methods**: Overnight HDEEG recordings were obtained in the patient during REM sleep, NREM sleep, wakefulness, and during a right facial motor seizure then compared to 10 controls. After preprocessing, SWA (i.e., delta power; 1–4 Hz) was calculated at each channel. Scalp level and source reconstruction analyses were computed. We assessed for statistical differences in maximum SWA between the patient and controls within REM sleep, NREM sleep, wakefulness, and seizure. Then, we completed an identical statistical comparison after first subtracting intrasubject REM sleep SWA from that of NREM sleep, wakefulness, and seizure SWA.

**Results**: The topographical analysis revealed greater left hemispheric SWA in the patient vs. controls in all vigilance states except REM sleep (which showed a right hemispheric maximum). Source space analysis revealed increased SWA in the left inferior frontal cortex during NREM sleep and wakefulness. Ictal data displayed poor source-space localization. Comparing each state to REM sleep enhanced localization accuracy; the most clearly localizing results were observed when subtracting REM sleep from wakefulness.

**Conclusion**: State-dependent SWA during NREM sleep and wakefulness may help to identify aspects of the potential epileptogenic zone. Future work in larger cohorts may assess the clinical value of sleep SWA to help presurgical planning.

## Introduction

Epilepsy proves drug-refractory in 20–40% of cases (Liu et al., 2015). In these patients, surgical resection represents the gold standard intervention (Ryvlin et al., 2014; Liu et al., 2015). Clinical 10-20 electroencephalography (EEG) is used pre-surgically to identify areas of the epileptogenic zone (Kahane et al., 2006; Lüders et al., 2006), although muscle artifacts and electrode number limit its localizing power (Lantz et al., 2003; Islam et al., 2016). High-density EEG (HDEEG; i.e., >64 electrodes) offers enhanced artifact rejection and source localization (Lantz et al., 2003; Puce and Hämäläinen, 2017). This technique is most commonly utilized to source-localize interictal spikes. However, in patients with focal epilepsy, the irritative zone identified through source localization of spikes does not necessarily match the ictal onset zone (IOZ; Strobbe et al., 2016; Verhoeven et al., 2018).

In a previous HDEEG study, we detected local increases in non-rapid eye movement (NREM) sleep slow-wave activity (SWA; i.e., delta power, 1–4 Hz) that colocalized with IOZs (Boly et al., 2017). In animal studies, epileptic activity induces synaptic potentiation (Debanne et al., 2006; Staley and Dudek, 2006). Because local sleep SWA reflects synaptic potentiation during wakefulness (Tononi and Cirelli, 2014), and the increased sleep SWA we observed was correlated with epileptic spike and seizure frequency (Boly et al., 2017), it likely reflected synaptic potentiation induced by epileptic activity in human brains. Epilepsy-induced increased SWA is reminiscent of task-dependent local SWA observed during wakefulness and sleep after learning (Siclari and Tononi, 2017)—suggesting that epileptic disease may hijack the normal mechanisms of synaptic plasticity (Boly et al., 2017).

While our previous findings were promising, not all patients in the study displayed NREM sleep SWA maximums that colocalized to their IOZ. One reason may be that we only assessed overall NREM sleep SWA, without considering state-dependence—for this manuscript, we use the term “state-dependent” to refer to how SWA varies across vigilance states, such as REM sleep, NREM sleep, wakefulness, or seizure. In neurological disease, focal SWA is a non-specific EEG finding, also occurring secondary to deafferentation in brain lesions caused by stroke or traumatic brain injury—in which case it may be independent of sleep-wakefulness processes (Dunkley et al., 2015; Rabiller et al., 2015; Rosanova et al., 2018). Given that REM sleep is thought to suppress epileptic activity (Shouse et al., 2000; Ng and Pavlova, 2013; Lambert et al., 2018; Kang et al., 2020), state-dependent SWA may more specifically localize epileptic foci, especially when comparing NREM sleep to REM sleep. This hypothesis is in line with our previous study where we reported no significant abnormality during REM sleep in patients’ HDEEG topographies but noted increases in SWA during NREM sleep compared to REM sleep (Boly et al., 2017). Consequently, we here aimed to evaluate the localizing value of state-dependent SWA during NREM sleep, wakefulness, and seizure—first, within each state in isolation, and then with each state initially subtracted by intrasubject REM sleep SWA.

The current study considers a patient with reflex epilepsy, providing an opportunity to test a proof-of-concept—that is, a pilot study to consider the feasibility of larger-scale analysis—for the localizing value of SWA across different vigilance states (Koepp et al., 2016). In the aforementioned previous study, we compared the location of HDEEG-detected local SWA to the location of IOZ, identified *via* the 10-20 clinical EEG montage in patients with focal epilepsy. The 10-20 EEG possesses limited spatiotemporal resolution. Also, non-lesional epilepsy often proves difficult to localize. The reflex epilepsy patient described here, although non-lesional, presented with a readily localizable ictal symptomatogenic zone due to her purely motor ictal semiology. Her seizures, triggered by eating, were characterized by stereotypic right facial twitching with fully preserved awareness, localizable to the left motor cortex. Ictal SPECT data was also available for the patient, which displayed an area of hyperperfusion in the left inferior frontal cortex. The case thus offered a chance to assess the localizing value of state-dependent SWA in focal epilepsy.

In this study, we statistically compared state-dependent SWA (i.e., delta power, 1–4 Hz) in a reflex epilepsy patient to 10 healthy controls utilizing 256-electrode HDEEG scalp level topographies and source reconstruction analyses. “Scalp level”, as utilized in this manuscript, refers to the topographic mapping of SWA (Nuwer, 1988). This approach provides a two-dimensional rendering of brain activity (Wang et al., 2017). “Source space” or “source reconstruction” analysis combined these data with a forward model and minimum norm plus smoothness priors to provide a three-dimensional location of SWA changes on the cortical surface (López et al., 2014; Michel and Brunet, 2019). Since recent literature emphasizes the presence of plasticity-induced local SWA not only during NREM sleep, but also during wakefulness (Tononi and Cirelli, 2014), we compared the patient’s SWA data to controls during REM sleep, NREM sleep, and wakefulness states, as well as during a seizure. Then, we subtracted each subjects’ REM sleep SWA from their own SWA during NREM sleep and wakefulness states, plus the seizure state for the patient, before running an identical analysis as above (in effect, utilizing the physiological observation that REM sleep inhibits epileptic activity). We hypothesized that maximum changes in state-dependent SWA (i.e., its variation across various vigilance states) in the patient vs. controls may colocalize with regions of the patient’s epileptogenic zone, especially when comparing other states to REM sleep.

## Materials and Methods

### Case Presentation

HDEEG data from a 61-year-old right-handed woman with drug-refractory reflex epilepsy was utilized for this study. She presented with right facial motor seizures caused by eating. The patient underwent EEG recording while at the University of Wisconsin’s Epilepsy Monitoring Unit (EMU). Clinical 10-20 EEG revealed left frontal interictal epileptic discharges (IED; Supplementary Figure 2). Several seizures were captured on 10-20 EEG but were uninterpretable due to movement artifacts. One seizure was recorded with HDEEG. An MRI during the patient’s EMU stay did not reveal structural lesions that correlated with her seizure semiology. A thorough clinical history of the patient may be found on page 9 of the Supplementary Materials.

The patient was initially referred to a general neurology clinic at age 45. She reported 5 years of 2-min episodes of dizziness, light-headedness, and flushes of warmth followed by chills, diaphoresis, and spells of “blank out.” MRI and EEG findings were nonspecific at that time. By age 49 she experienced a fine tongue tremor and dysarthria as well as facial twitching episodes, as the above-described semiology waned. Repeat MRI imaging was unremarkable. However, an ictal SPECT co-registered to MRI showed increased perfusion in the left inferior frontal lobe (unfortunately, only the official read found within the patient’s medical record was available, i.e., the images were not accessible, as they were obtained at an outside institution).

Right facial seizures continued unabated 11 years status post initial presentation despite multiple anti-seizure drug (ASD) trials (including lamotrigine, zonisamide, levetiracetam, carbamazepine, topiramate, oxcarbazepine, and phenytoin). Events occurred up to twice daily and now included contraction of the right sternocleidomastoid muscle. At that point, the patient mentioned that her seizures were triggered by chewing food with the right side of her mouth. Fifteen years after the initial presentation she underwent the inpatient EMU stay, during which the present HDEEG data was recorded. Thereafter, seizures continued, as did trials of ASDs. The patient relocated and was lost to follow-up 18 years after the first presentation.

### HDEEG Monitoring and Analyses

The Institutional Review Board at the University of Wisconsin-Madison approved our study methods. A single overnight recording with a 256-electrode EEG net (EGI, Electrical Geodesics Inc.) was conducted on the patient and 10 healthy control volunteers. Controls from an unrelated study were utilized, having been recruited at the Wisconsin Sleep Laboratory; each was free of neurological disease, sleep disorders, and medications. The patient and each control gave written informed consent before participation in the EEG procedure.

EEG data preprocessing was completed similarly to previous studies (Boly et al., 2017). For each subject, epochs of interest were extracted: an approximately 5-min epoch of clean REM sleep and N3 NREM sleep, a 2–3-min epoch of wakefulness; and an additional 70-s epoch of clinical ictal activity was extracted for the patient (full clinical seizure epoch). A certified epileptologist (MB) identified IEDs, which were then removed from the data. Using Matlab software, we filtered data from 1 to 40 Hz for NREM sleep and REM sleep epochs using EEGlab default FIR filter (as in Boly et al., 2017) and from 1 to 25 Hz for wakefulness and seizure epochs (to reduce muscle activity contribution). We first rejected and interpolated bad channels—for the patient this included seven channels during REM sleep, four for NREM sleep, five during wakefulness, and 75 during a seizure. Then, noisy epochs were rejected before conducting independent component analysis (runica EEGlab plugin, separately for each condition) to remove eye movement, cardiac, and muscle artifacts. All statistics utilized average-referenced EEG data. For all scalp level analyses, the root mean square of delta band activity (1–4 Hz) was computed to estimate SWA at each channel separately for REM sleep, NREM sleep, wakefulness, and seizure epochs; topographical images were generated to perform scalp statistics on the results using Statistical Parametric Mapping (SPM) software^1^. We also conducted a source reconstruction of pre-processed HDEEG data using Brainstorm software^2^ (Tadel et al., 2011). We used realistic template head models with the boundary element method plus minimum norm and smoothness (LORETA) priors. We then estimated source-space SWA using Welch’s power spectral density function (MATLAB pwelch).

To conduct statistical analyses, scalp topographical values of SWA were converted to 2D images, and source space SWA values were converted into gifti images (Kilner and Friston, 2010; Boly et al., 2017). Within-subject spatial normalization of SWA was conducted *via* *z*-scoring. SPM software was then utilized to run *t*-tests on consistent SWA differences between the patient and each control (on the difference images between the patient and each control), using a random-effects approach (Siegel et al., 2014). For both scalp topographies and source space models, the consistency of SWA differences between the patient and each control were assessed within isolated REM sleep, NREM sleep, and wakefulness vigilance states; for the patient’s ictal analysis, the wakefulness SWA of each control was compared to the patient’s seizure. Finally, an identical statistical analysis was completed using images wherein each participant’s REM sleep SWA was first subtracted from their NREM sleep SWA, wakefulness SWA, or seizure SWA (in the case of the patient); that is, before re-assessing for consistent SWA differences between the patient and each control within each vigilance state (excluding REM sleep). All results were thresholded at cluster-level or peak-level *p* < 0.05 corrected for multiple spatial comparisons using a family-wise error rate as implemented in SPM.

## Results

### Demographics and Sleep Architecture

Supplementary Table 1 summarizes the control group’s demographics. The control group was age-matched with the patient. Patient and control sleep architecture data for the single-overnight HDEEG recording can also be found in this table. Sleep onset latency (SOL) and REM sleep latency (REML) was higher in the patient as compared to the control group average. The patient also had a higher proportion of N3 stage NREM sleep and a lower proportion of REM sleep (as compared to total sleep time) than controls. Other sleep architecture parameters were similar to controls.

### Scalp Level Results

**Figure 1:**
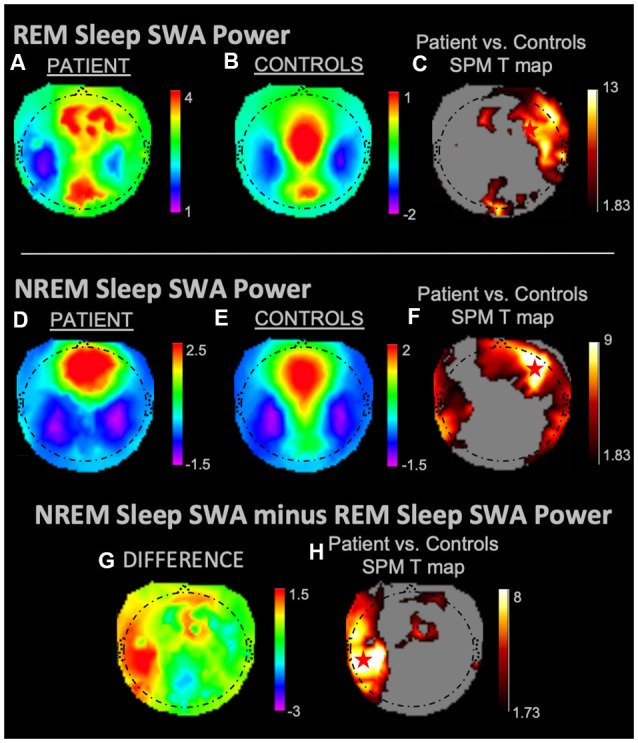
Slow-wave activity (SWA) power topographies for the patient and controls during REM sleep and non-rapid eye movement (NREM) sleep taken in isolation, and for intrasubject NREM sleep subtracted by REM sleep. Red stars denote SWA maximums. REM sleep and NREM sleep in isolation displayed SWA maxima in the right frontal areas. We observed a left fronto-temporal NREM sleep SWA maximum as referenced to intrasubject REM sleep SWA. **(A)** REM sleep mean SWA power for the patient. **(B)** REM sleep mean SWA power for controls. **(C)** Statistical Parametric Mapping (SPM) T map for the statistical difference between patient and controls REM sleep SWA; the red star shows the SWA maximum. **(D)** NREM sleep mean SWA power for the patient. **(E)** NREM sleep mean SWA power for controls. **(F)** SPM T map for the statistical difference between patient and controls NREM sleep SWA; the red star shows the SWA maximum. **(G)** Difference between patient and controls NREM sleep SWA subtracted by REM sleep SWA. **(H)** SPM T map for the difference between patient and controls SWA power during NREM sleep subtracted by REM sleep SWA; the red star shows the SWA maximum. Each T map pictured above is thresholded for a display of uncorrected *p* < 0.05. The rainbow color scale is utilized for regression slope values and the hot color scale for *t*-values.

**Figure 2:**
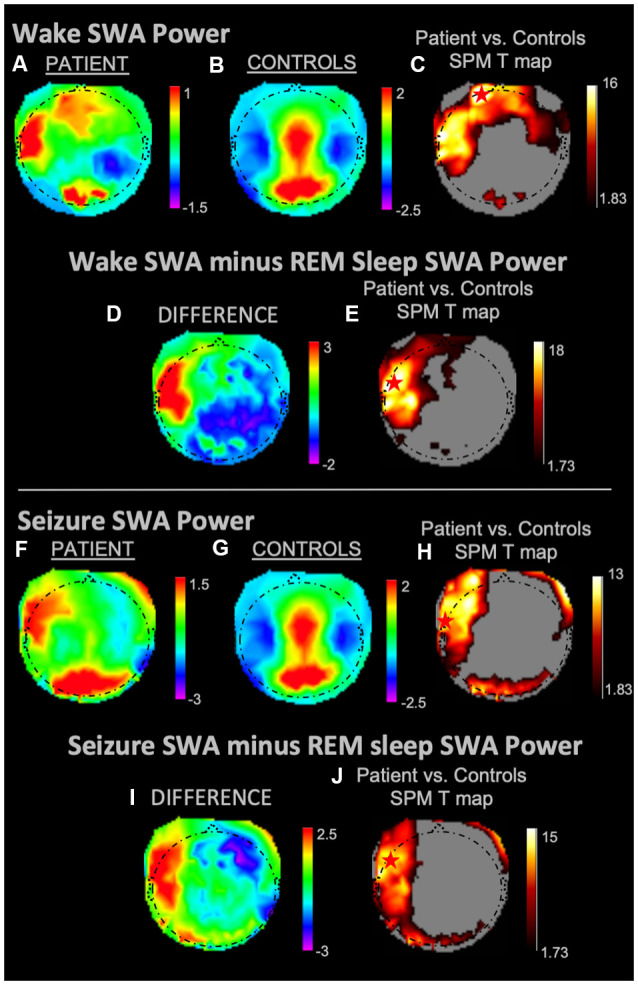
SWA power topographies for the patient and controls during wakefulness and seizure states, both in isolation and when subtracted by REM sleep. Red stars denote SWA maximums. Wakefulness SWA analysis showed a left frontal maximum; when referenced to REM sleep, wakefulness SWA localized to the left fronto-temporal region. The seizure state displayed an SWA maximum in the left temporal region. We observed a left fronto-temporal region SWA maximum in the seizure state when referenced to intrasubject REM sleep SWA. **(A)** Wakefulness mean SWA power for the patient. **(B)** Wakefulness mean SWA power for controls. **(C)** SPM T map for the statistical difference between patient and controls wakefulness SWA; the red star shows the SWA maximum. **(D)** Difference between patient and controls SWA power during wakefulness subtracted by REM sleep SWA. **(E)** SPM T map for the difference between patient and controls SWA power during wakefulness subtracted by REM sleep SWA; the red star shows the SWA maximum. **(F)** Seizure mean SWA power for the patient. **(G)** Wakefulness mean SWA power for controls. **(H)** SPM T map for the statistical difference in SWA power between patient seizure and controls wakefulness; the red star shows the SWA maximum. **(I)** The difference in mean SWA power between patient seizure and wakefulness of controls, both subtracted REM sleep SWA. **(J)** SPM T map for patient seizure and controls wakefulness subtracted by REM sleep SWA; the red star shows the SWA maximum. Each T map pictured above is thresholded for a display of uncorrected *p* < 0.05. The rainbow color scale is utilized for regression slope values and the hot color scale for *t*-values.

During REM sleep, SWA maximally increased in the right frontal area in the patient vs. controls (Figures 1A–C). During NREM sleep, SWA increased bilaterally in front-temporal regions, with a maximum in the right frontal area (Figures 1D–F). When subtracting each participant’s REM sleep from their NREM sleep before completing an identical statistical analysis (Figures 1G,H), we observed focal increases in SWA within the left central region. For wakefulness, SWA increased maximally in a left centrotemporal region (Figures 2A–C)—a finding confirmed when comparing wakefulness to REM sleep as above (Figures 2D,E). Scalp-level topography during the patient’s seizure compared to the wakefulness of the controls (Figures 2F–H) revealed a maximum SWA increase in the left centrotemporal region. For seizure (i.e., the patient’s ictal period vs. controls’ wakefulness) compared to REM sleep (Figures 2I,J), the SWA maximal increase also localized to the left centrotemporal area. Supplementary Figure 1 shows a topographic rendering of the patient’s ictal data across wider frequency bands beyond SWA, with a special aim towards differentiating lower vs. higher frequency SWA. Supplementary Table 2 displays scalp topographic coordinates and statistical values for maximal differences in SWA in the patient vs. controls across all vigilance states.

### Source Reconstruction Results

**Figure 3:**
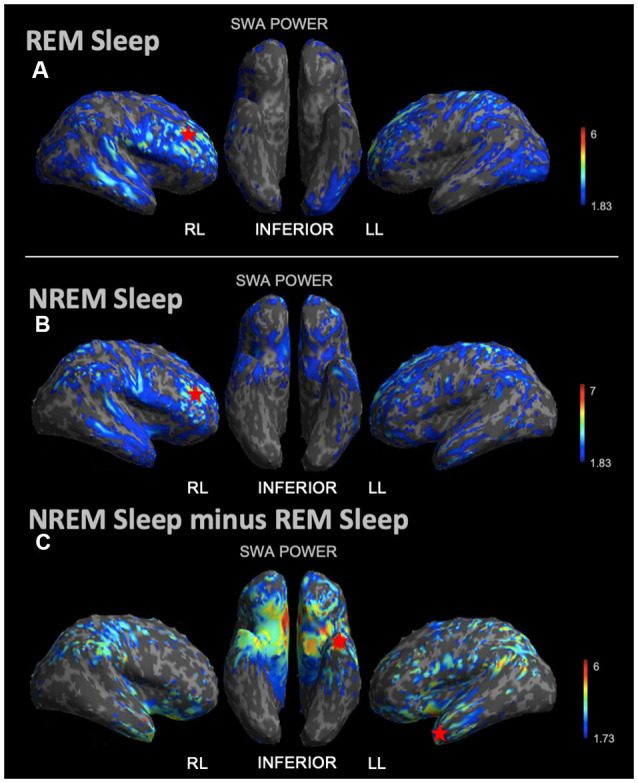
Source space reconstruction of SWA power for the patient vs. controls during NREM sleep and REM sleep taken in isolation, and for NREM sleep subtracted by REM sleep. Red stars denote SWA maximums. We observed NREM sleep and REM sleep SWA maxima within the right frontal lobe. SWA maxima localized to the left temporal lobe when intrasubject NREM sleep SWA was subtracted by REM sleep SWA before statistical analysis. **(A)** Patient REM sleep SWA power subtracted by controls REM sleep SWA power; the red star shows the SWA maximum. **(B)** Patient SWA power subtracted by controls SWA power during NREM sleep; the red star shows the SWA maximum. **(C)** Patient vs. controls comparison for NREM sleep SWA power subtracted by REM sleep SWA power; the red star shows the SWA maximum. Each T map pictured above is thresholded for a display of uncorrected *p* < 0.05. The jet color scale indicates *t*-values. Abbreviations: RL = right lateral; LL = left lateral.

**Figure 4:**
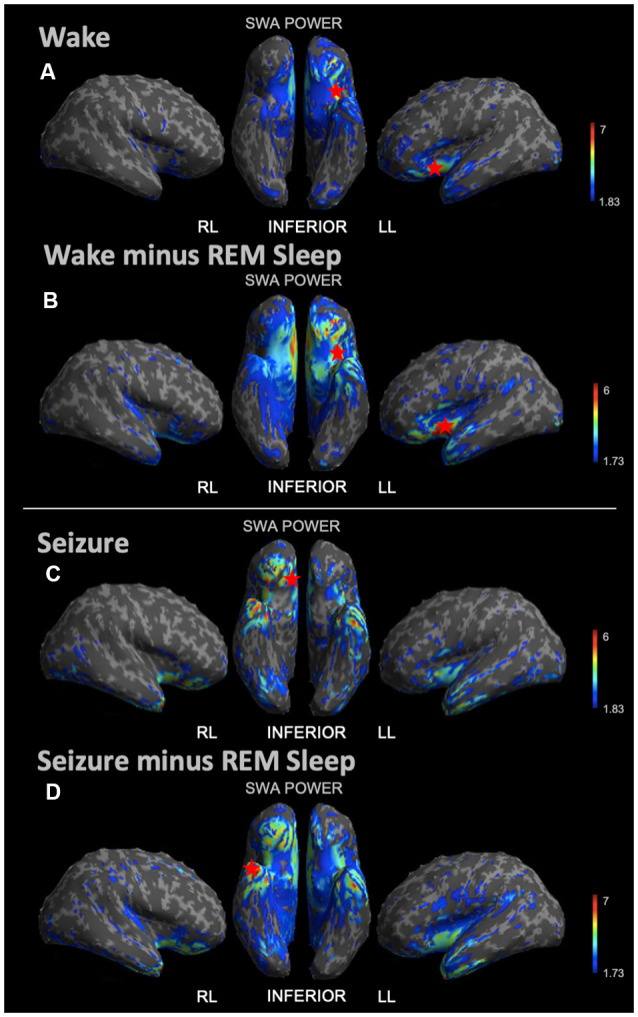
Source space reconstruction of SWA power for the patient vs. controls during wake and seizure states, both taken in isolation and when subtracted by REM sleep. Red stars denote SWA maximums. We observed the SWA maximum during wakefulness in the left inferior frontal lobe. We observed the SWA maximum during seizure within the right limbic area. When these states were first subtracted by intrasubject REM sleep SWA before statistical re-analysis, we again observed the wakefulness SWA maximum in the left inferior frontal lobe, albeit with a higher power; and for the seizure state, we observed the SWA maximum within the right temporal lobe. **(A)** Patient vs. controls mean SWA power during wakefulness; the red star shows the SWA maximum. **(B)** SWA power in patient vs. controls for wakefulness subtracted by REM sleep; the red star shows the SWA maximum. **(C)** Patient seizure SWA power vs. controls wakefulness SWA power. **(D)** Patient seizure vs. controls wakefulness SWA power subtracted by REM sleep SWA power; the red star shows the SWA maximum. Each T map pictured above is thresholded for a display of uncorrected *p* < 0.05. The jet color scale indicates *t*-values. Abbreviations: RL = right lateral; LL = left lateral.

During REM sleep, we noted an SWA maximum in the right frontal lobe (Figure 3A). Source reconstruction demonstrated a maximum SWA increase in the right frontal cortex during NREM sleep (Figure 3B). When the patient’s and each control’s NREM sleep SWA was first subtracted by their REM sleep SWA (Figure 3C), we found a maximum change in SWA within the left temporal region after running an identical statistical analysis. Wakefulness source-space data (Figure 4A), both in isolation and when compared to REM sleep, as above (Figure 4B), had the best localizing value with an SWA maximum increase found in the left inferior frontal cortex. Ictal source reconstruction data (Figure 4C) revealed bilateral limbic increases in SWA; comparing this data to REM sleep revealed a right temporal SWA maximum (Figure 4D). Supplementary Figure 1 shows the source space rendering of IEDs captured within NREM sleep during HDEEG monitoring. Supplementary Table 3 reports cortical coordinates and statistical values for maximal SWA changes when comparing the patient to controls across all vigilance states.

## Discussion

Here we present HDEEG analysis of SWA across different vigilance states in a patient with reflex epilepsy compared to 10 controls. Our results serve as a proof-of-concept, suggesting that validation studies may be feasible, for the localizing value of state-dependent increases in SWA to identify components of the epileptogenic zone. At the scalp level and in source space we observed no difference in REM sleep SWA between the patient and controls. Altogether, we observed localizing value for both scalp-level and source space SWA during NREM sleep and wakefulness states, with peak accuracy achieved when comparing states to REM sleep. Interestingly, the most robust SWA localizing power in source space was observed when comparing wakefulness to REM sleep.

### Sleep SWA

Although our previous work in focal epilepsy patients suggested some localizing value of scalp-level NREM sleep SWA increases (Boly et al., 2017), accurate localization power was present in only 11/15 patients. Here we performed a proof-of-concept analysis for the localization value of state-dependent SWA. At the scalp level, we observed a right frontal maximum during both REM sleep and NREM sleep, which did not correctly lateralize the IOZ. Correct scalp lateralization was however noted when we compared NREM sleep to REM sleep. Similarly, source localization of NREM sleep SWA taken in isolation did not correctly localize the IOZ. Once referenced to REM sleep, localization power improved.

These NREM sleep findings are in line with our previous observation of bilateral SWA changes during NREM sleep (Boly et al., 2017), which may be indicative of more widespread plastic changes occurring due to the ictal involvement of a bilateral epileptic network (Blumenfeld, 2014). What’s more, our results may be hindered by a lack of subject-specific head models utilized for source reconstruction (Klamer et al., 2015). These findings also match previous observations from our group (Boly et al., 2017), and others (Shouse et al., 2000; Ng and Pavlova, 2013; Frauscher et al., 2016; Lambert et al., 2018), that REM sleep inhibits not only overall SWA but more specifically SWA related to epileptic activity. REM sleep is a state characterized by EEG desynchronization, as demonstrated by decreased power in frequencies <30 Hz (Frauscher et al., 2016), probably mediated by cholinergic neurotransmission arising from subcortical inputs. This generalized tendency toward low voltage fast activity may favor the break-up of synchrony within epileptic networks (Shouse et al., 2000; Ng and Pavlova, 2013; Lambert et al., 2018; Kang et al., 2020). Overall, our results emphasize the importance of assessing local SWA across multiple vigilance states (i.e., comparing NREM sleep to REM sleep), suggesting that the opportunistic utilization of REM sleep SWA in the setting of epilepsy may assist to accentuate epilepsy-induced SWA maximums.

### Wakefulness SWA

In contrast to NREM sleep, scalp level SWA during the patient’s waking state was lateralizing for the IOZ even when considered in isolation. When subtracting individual REM sleep SWA from wakefulness SWA before re-running an identical statistical analysis, we again observed enhanced localization, with more focal results pointing to the left inferior frontal gyrus—which correlates with the patient’s ictal SPECT imaging from an outside institution 15 years prior. These findings reiterate the added value of considering state-dependent SWA for epileptic focus identification. The powerful localization value of wakefulness SWA, both in isolation and when referenced to REM sleep, is especially interesting in the context of recent experiments documenting the presence of local increases in SWA during wakefulness after normal learning (Huber et al., 2004). For example, our group has shown that increased SWA during wakefulness localizes to task-dependent cortical regions during extended wakefulness (Hung et al., 2013); e.g., language task learning increased SWA in the left frontal cortex, and motor learning in the contralateral motor cortex (Siclari and Tononi, 2017). Because animal models suggest that epileptic activity leads to synaptic potentiation (Tononi and Cirelli, 2014), and increased synaptic strength saturation leads to the emergence of local sleep SWA during wakefulness (Vyazovskiy et al., 2011), plastic changes induced by seizures and interictal spikes may also induce local sleep during wakefulness within the epileptic focus. This phenomenon could be protective at some level, by decreasing the local propensity towards ictal processes. However, it may also prevent the epileptic cortex from participating in normal task functions even during wakefulness. Of note, somatostatin-positive interneurons (thought to correspond to Martinotti-cells) generate local SWA in mice (Funk et al., 2017) and are themselves potentiated by excitatory tone (Kroon et al., 2019). We, therefore, speculate that these interneurons may be involved in the generation of SWA during wakefulness in the hyperexcitable epileptic cortex, even if subcortical neuromodulatory tone favors the presence of wakefulness rhythms in the rest of the cortex. Such a hypothesis would however require testing in animal models.

### Ictal SWA

Maximum scalp level increases in SWA during the seizure, when compared to control wake images, were correctly lateralized (in the left temporal lobe). Comparing this state to intrasubject REM sleep, before completing an identical statistical analysis, provided enhanced localization (maximum in SWA observed in the left frontal lobe). These results again underscore the value of analyzing SWA changes across vigilance states. However, after source reconstruction, the maximum in SWA during the patient’s seizure did not co-localize with the ictal symptomatogenic zone. This was true when ictal SWA was taken in isolation and when first subtracted by REM sleep SWA. This finding may be because our EEG source reconstruction method did not incorporate an explicit noise model, and thus had difficulties dealing with the residual noise likely present in the ictal EEG dataset despite our artifact removal attempts. Further, we utilized default head models for the patient and all controls, instead of importing specific MRI data for each individual. This approach has been shown to decrease the precision of HDEEG localization (Klamer et al., 2015). Alternatively, our findings may again hint at larger recruitment of neuronal networks by SWA during the ictal state, extending beyond the epileptic focus itself (Blumenfeld, 2014).

### Limitations

Drawbacks to collecting HDEEG data within the inpatient epilepsy monitoring unit include potential sleep disruptions. This may be reflected by the patient’s higher SOL, decreased total REM sleep time, and increased REML. However, these changes in sleep architecture were also reported in outpatient recordings in other epileptic patients and are not expected to alter the spatial topography of SWA within each sleep stage (Foldvary-Schaefer and Grigg-Damberger, 2006).

Although independent component analysis has become state-of-the-art for the preprocessing of HDEEG studies (Strobbe et al., 2016), there remains some user-dependence on this method. Such user-dependence is most likely to affect the pre-processing of data collected during wakefulness and ictal states, which are more affected by movement artifacts. Future cohort studies in larger populations will permit sub-group analysis for epilepsies of different localization and severity, exploration of neuropsychological associations, and further assessment of the reproducibility in localization accuracy for state-dependent increases in SWA.

Source space analysis in this study was limited given that we utilized template head models for the patient and all controls. HDEEG localization results have been shown to vary by up 2 cm (Klamer et al., 2015). Thus, discrepancies in the patient’s semiologic presentation may reflect varying aspects of her epileptogenic zone, wherein her semiology differs from her IOZ (as identified by our SWA localization and outside hospital SPECT analysis), or imprecise localization accuracy due to methodologic limitations. Future studies may aim to incorporate specific modeling parameters to enhance accuracy and thereby clarify such concerns.

Given that the patient has been lost to follow up, and that much of her medical care was obtained from outside of our institution, we lack comprehensive radiological imaging to include in this study. A larger-scale analysis probing the utility of SWA localization power in epilepsy patients might utilize advanced imaging such as FDG-PET, ictal SPECT, and comparative MRI analysis—all of which may improve spatial resolution and better characterize components of the epileptogenic zone. Finally, intracranial recording studies may further assess the spatial resolution of the ictal seizure localization provided by state-dependent SWA changes and correlate them with surgical outcomes.

### Clinical Promise

State-dependent SWA analysis may constitute a new and promising clinical tool to diagnose and localize focal epilepsy. HDEEG studies across vigilance states may provide meaningful methods to map focal epileptic networks while reducing the need to capture seizures. With further validation, these methods may assist with presurgical planning in epilepsy patients.

## Data Availability Statement

The raw data supporting the conclusions of this article will be made available by the authors, without undue reservation.

## Ethics Statement

The studies involving human participants were reviewed and approved by The Institutional Review Board at the University of Wisconsin-Madison, Madison, WI, USA. The patients/participants provided their written informed consent to participate in this study. Written informed consent was obtained from the individual(s) for the publication of any potentially identifiable images or data included in this article.

## Author Contributions

EM, GT, and MB designed the study. EM and MB conducted EEG signal preprocessing and statistical analysis. EM and MB drafted the manuscript for intellectual content, plus edited and formatted the manuscript for submission. MB, RV, BJ, GF, EJ, TB, MA, and AM contributed to data acquisition. Each author critically reviewed the manuscript. All authors contributed to the article and approved the submitted version.

## Conflict of Interest

The authors declare that the research was conducted in the absence of any commercial or financial relationships that could be construed as a potential conflict of interest.

## Footnotes

^1^www.fil.ion.ucl.ac.uk/spm

^2^https://www.nitrc.org/projects/bst/
