# Supplementary material for: Local Sleep Slow-Wave Activity Colocalizes With the Ictal Symptomatogenic Zone in a Patient With Reflex Epilepsy: A High-Density EEG Study
Source: Front Syst Neurosci. 2020 Oct 21;14:549309. doi: 10.3389/fnsys.2020.549309 (PMC7609881; doi:10.3389/fnsys.2020.549309)
Supplement: Supplementary file 1 [file Table_1.DOCX]

**Supplementary Table 1. Demographic and sleep architecture data for patient and controls.**

| **Study Participant** | **Sex** | **Age (years)** | **SOL (min)** | **WASO (min)** | **TST (min)** | **N1, % TST** | **N2, % TST** | **N3, % TST** | **REM, % TST** | **REML (min)** | **SE, %** |
| --- | --- | --- | --- | --- | --- | --- | --- | --- | --- | --- | --- |
| Patient | F | 61 | 33 | 28 | 443 | 29 | 36 | 26 | 9 | 307 | 88 |
| Control Group Mean (SD) |  | 48 (13) | 15 (6) | 34 (18) | 408 (22) | 19 (10) | 43 (9) | 16 (6) | 22 (4) | 89 (36) | 89 (4) |
| Control 1 | F | 61 | 30 | 27 | 425 | 41 | 29 | 13 | 17 | 168 | 88 |
| Control 2 | M | 65 | 17 | 67 | 385 | 17 | 47 | 11 | 25 | 105 | 82 |
| Control 3 | F | 46 | 13 | 23 | 447 | 18 | 46 | 12 | 24 | 85 | 93 |
| Control 4 | F | 36 | 16 | 42 | 436 | 16 | 49 | 9 | 26 | 66 | 88 |
| Control 5 | F | 59 | 8 | 46 | 411 | 9 | 35 | 27 | 29 | 49 | 88 |
| Control 6 | F | 42 | 18 | 13 | 404 | 27 | 35 | 19 | 19 | 74 | 93 |
| Control 7 | F | 62 | 8 | 52 | 386 | 18 | 40 | 22 | 20 | 82 | 87 |
| Control 8 | F | 33 | 12 | 14 | 404 | 9 | 52 | 19 | 20 | 128 | 94 |
| Control 9 | F | 30 | 16 | 17 | 388 | 9 | 58 | 10 | 23 | 72 | 92 |
| Control 10 | M | 48 | 10 | 38 | 397 | 27 | 41 | 17 | 15 | 61 | 89 |

**Abbreviations**: N1 = stage 1 non-rapid eye movement sleep; N2 = stage 2 non-rapid eye movement sleep; N3 = stage 3 non-rapid eye movement sleep; REM = rapid eye movement sleep; REML = REM sleep latency; SD = standard deviation; SE = sleep efficiency; SOL = sleep onset latency; TST = total sleep time; WASO = wake after sleep onset.

**Supplementary Table 2. Scalp-level state-dependent SWA power maximums for left and right hemispheres.** Coordinates expressed as distance from a midline reference point.

| **Left Hemisphere SWA Power Maximums** | | | | | **Right Hemisphere SWA Power Maximums** | | | | |
| --- | --- | --- | --- | --- | --- | --- | --- | --- | --- |
| **Scalp Area** | **Coord. (x, y)** | **Z value** | **P Value corrected** | **P Value uncorrected** | **Scalp Area** | **Coord. (x, y)** | **Z value** | **P Value corrected** | **P Value uncorrected** |
| **NREM Sleep SWA** | | | | | | | | | |
| Left Lateral Temporal | -60 -46 | 3.87 | 0.035^v^  0.001^c^ | <0.001^v,c^ | Right Central Frontal Pole | 34 37 | 5.59 | <0.001^v,c^ | <0.001^v,c^ |
| Left Lateral Temporal | -62 -38 | 3.85 | 0.038 | <0.001 | Right Central Frontal | 34 24 | 4.60 | 0.003 | <0.001 |
| Left Lateral Frontal | -66 -13 | 3.38 | 0.139 | <0.001 | Right Central Frontal | 32 13 | 4.45 | 0.012 | <0.001 |
| **NREM Sleep SWA minus REM Sleep SWA** | | | | | | | | | |
| Left Lateral Central | -32 -22 | 6.10 | <0.001^v,c^ | <0.001^v,c^ | Right Medial Central | 6 5 | 3.92 | 0.019^v^  0.278^c^ | <0.001^v^  0.087^c^ |
| Left Lateral Temporal | -53 -33 | 5.82 | <0.001 | <0.001 | Right Medial Central | 17 8 | 2.96 | 0.289 | 0.002 |
| Left Medial Temporal | -38 -27 | 5.55 | 0.002 | <0.001 | Left Medial Central | -13 -9 | 2.17 | 0.833 | 0.015 |
| **REM Sleep SWA** | | | | | | | | | |
| Left Medial Occipital | -2 -89 | 4.82 | 0.002^v^  0.081^c^ | <0.001^v^  0.020^c^ | Right Lateral Frontal | 51 29 | 5.24 | <0.001^v,c^ | <0.001^v,c^ |
| Left Medial Occipital | -8 -78 | 4.74 | 0.002 | <0.001 | Right Lateral Temporal | 62 -19 | 5.11 | <0.001 | <0.001 |
| Left Medial Parietal | -8 -65 | 3.42 | 0.071 | <0.001 | Right Medial Temporal | 45 -9 | 5.06 | 0.001 | <0.001 |
| **Seizure SWA** | | | | | | | | | |
| Left Lateral Temporal | -60 2 | 5.62 | <0.001^v,c^ | <0.001^v,c^ | Right Lateral Frontal | 62 37 | 5.48 | <0.001^v^  0.020^c^ | <0.001^v^  0.005^c^ |
| Left Central Frontal Pole | -28 56 | 5.36 | <0.001 | <0.001 | Right Lateral Frontal Pole | 49 56 | 5.02 | 0.001 | <0.001 |
| Left Central Frontal | -34 26 | 5.28 | <0.001 | <0.001 | Right Lateral Frontal Pole | 38 64 | 4.63 | 0.003 | <0.001 |
| **Seizure SWA minus REM Sleep SWA** | | | | | | | | | |
| Left Lateral Temporal | -60 2 | 7.43 | <0.001^v,c^ | <0.001^v,c^ | Right Lateral Frontal Pole | 60 45 | 6.55 | <0.001^v^  0.197^c^ | <0.001^v^  0.057^c^ |
| Left Medial Temporal | -36 -14 | 6.97 | <0.001 | <0.001 | Right Lateral Frontal Pole | 62 37 | 6.53 | <0.001 | <0.001 |
| Left Lateral Frontal | -45 13 | 6.95 | <0.001 | <0.001 | Right Lateral Frontal Pole | 49 56 | 5.96 | <0.001 | <0.001 |
| **Wake SWA** | | | | | | | | | |
| Left Lateral Frontal Pole | -23 53 | 5.89 | <0.001^v,c^ | <0.001^v,c^ | Right Medial Occipital | 19 70 | 3.66 | <0.001^v^  0.947^c^ | <0.001^v^  0.711^c^ |
| Left Lateral Temporal | -60 2 | 5.77 | <0.001 | <0.001 | No additional right hemisphere maximums. | | | | |
| Left Lateral Temporal | -60 -19 | 5.58 | <0.001 | <0.001 |  |  |  |  |  |
| **Wake SWA minus REM Sleep SWA** | | | | | | | | | |
| Left Lateral Temporal | -60 2 | 7.62 | <0.001^v,c^ | <0.001^v,c^ | Right Medial Occipital | 2 -76 | 2.18 | 0.841^v^  0.962^c^ | 0.015^v^  0.847^c^ |
| Left Lateral Temporal | -60 -19 | 7.52 | <0.001 | <0.001 | Right Medial Occipital | 19 -70 | 1.71 | 0.973^v^  0.977^c^ | 0.043^v^  0.982^c^ |
| Left Medial Temporal | -34 -14 | 7.26 | <0.001 | <0.001 | No additional right hemisphere maximums. | | | | |

**Abbreviations**: SWA = slow wave activity; Coord. = coordinates; REM = rapid eye movement; NREM = non-rapid eye movement

^v^ voxel-level; ^c^ cluster-level

**Supplementary Table 3. Source reconstruction of state-dependent SWA power maximums.** Coordinates expressed as distance from the anterior commissure in MNI space.

| **Location** | **BA** | **Cortical Region** | **Coord.**  **(x, y, z)** | **Z value** | **P value**  **corrected** | **P value**  **uncorrected** |
| --- | --- | --- | --- | --- | --- | --- |
| **NREM Sleep SWA** | | | | | | |
| **Left** | 5 | Left postcentral gyrus | -27 -38 70 | 5.82 | <0.001^v,c^ | <0.001^v,c^ |
|  | 5 | Left postcentral gyrus | -28 -55 71 | 5.62 | <0.001 | <0.001 |
|  | 5 | Left postcentral gyrus | -19 -69 67 | 5.59 | <0.001 | <0.001 |
| **Right** | 10 | Right superior frontal gyrus | 41 51 22 | 6.29 | <0.001^v,c^ | <0.001^v,c^ |
|  | 9 | Right middle frontal gyrus | 42 39 34 | 6.10 | <0.001 | <0.001 |
|  | 7 | Right superior parietal lobule | 43 -63 57 | 6.03 | <0.001 | <0.001 |
| **Frontal** | No significant frontal lobe maximums. | | | | | |
| **NREM Sleep SWA minus REM Sleep SWA** | | | | | | |
| **Left** | 20 | Left middle temporal gyrus | -44 0 -40 | 6.05 | <0.001^v^  1.000^c^ | <0.001^v^  0.549^c^ |
|  | 20 | Left inferior temporal gyrus | -62 -12 -32 | 5.97 | <0.001^v,c^ | <0.001^v,c^ |
|  | 20 | Left inferior temporal gyrus | -51 -7 -32 | 5.96 | <0.001 | <0.001 |
| **Right** | 25 | Right subcallosal frontal gyrus | 1 10 -15 | 5.89 | <0.001^v,c^ | <0.001^v,c^ |
|  | 11 | Right orbitofrontal gyrus | 1 14 -25 | 5.77 | <0.001 | <0.001 |
|  | 25 | Right subcallosal frontal gyrus | 1 18 -10 | 5.72 | <0.001 | <0.001 |
| **Frontal** | 13 | Left inferior frontal gyrus | -41 21 9 | 5.47 | <0.001^v^  0.001^c^ | <0.001^v,c^ |
| **REM Sleep SWA** | | | | | | |
| **Left** | 10 | Left superior frontal gyrus | -33 | 6.97 | <0.001^v,c^ | <0.001^v,c^ |
|  | 10 | Left superior frontal gyrus | -16 | 6.85 | <0.001 | <0.001 |
|  | 10 | Left superior frontal gyrus | -29 | 6.64 | <0.001 | <0.001 |
| **Right** | 10 | Right middle frontal gyrus | 41 | 6.49 | <0.001^v,c^ | <0.001^v,c^ |
|  | 6 | Right precentral gyrus | 60 | 6.36 | <0.001 | <0.001 |
|  | 9 | Right middle frontal gyrus | 43 | 6.17 | <0.001 | <0.001 |
| **Frontal** | No significant frontal lobe maximums. | | | | | |
| **Seizure SWA** | | | | | | |
| **Left** | 20 | Left limbic uncus | -26 0 -45 | 5.46 | <0.001^v,c^ | <0.001^v,c^ |
|  | 20 | Left inferior temporal gyrus | -52 -8 -46 | 5.32 | 0.001 | <0.001 |
|  | 20 | Left inferior temporal gyrus | -57 -13 -42 | 5.07 | 0.003 | <0.001 |
| **Right** | 38 | Right limbic uncus | 16 1 -25 | 5.52 | <0.001^v^  0.217^c^ | <0.001^v^  0.002^c^ |
|  | Amg | Right limbic uncus | 28 -1 -23 | 5.41 | <0.001 | <0.001 |
|  | 20 | Right limbic uncus | 34 -5 -28 | 5.18 | 0.002 | <0.001 |
| **Frontal** | 47 | Left inferior frontal gyrus | -44 16 -8 | 5.01 | 0.004 | <0.001 |
| **Seizure SWA minus REM Sleep SWA** | | | | | | |
| **Left** | 20 | Left inferior temporal gyrus | -52 -8 -46 | 6.85 | <0.001^v,c^ | <0.001^v,c^ |
|  | 20 | Left limbic uncus | -26 0 -45 | 6.63 | <0.001 | <0.001 |
|  | 38 | Left limbic uncus | -21 6 -35 | 6.32 | <0.001 | <0.001 |
| **Right** | 38 | Right inferior temporal gyrus | 36 7 -49 | 7.10 | <0.001^v,c^ | <0.001^v,c^ |
|  | 38 | Right inferior temporal gyrus | 44 15 -46 | 7.03 | <0.001 | <0.001 |
|  | 20 | Right limbic uncus | 28 0 -39 | 6.89 | <0.001 | <0.001 |
| **Frontal** | No significant frontal lobe maximums. | | | | | |
| **Wake SWA** | | | | | | |
| **Left** | 11 | Left middle frontal gyrus | -27 38 -13 | 6.47 | <0.001^v,c^ | <0.001^v,c^ |
|  | 11 | Left orbitofrontal gyrus | -10 12 -20 | 6.11 | <0.001 | <0.001 |
|  | 38 | Left superior temporal gyrus | -30 11 -22 | 6.08 | <0.001 | <0.001 |
| **Right** | 25 | Right subcallosal frontal gyrus | 1 8 -11 | 5.46 | <0.001^v,c^ | <0.001^v,c^ |
|  | 25 | Right subcallosal frontal gyrus | 1 18 -11 | 5.44 | <0.001 | <0.001 |
|  | 11 | Right orbitofrontal gyrus | 2 12 -21 | 5.21 | 0.001 | <0.001 |
| **Frontal** | No significant frontal loabe maximums. | | | | | |
| **Wake SWA minus REM Sleep SWA** | | | | | | |
| **Left** | 25 | Left medial frontal gyrus | -12 20 -16 | 7.40 | <0.001^v,c^ | <0.001^v,c^ |
|  | Amg | Left parahippocampal gyrus | -30 0 -20 | 7.32 | <0.001 | <0.001 |
| **Right** | 25 | Right subcallosal frontal gyrus | 1 8 -11 | 6.93 | <0.001^v,c^ | <0.001^v,c^ |
|  | 25 | Right subcallosal frontal gyrus | 1 18 -11 | 6.76 | <0.001 | <0.001 |
|  | 11 | Midline orbitofrontal gyrus | 0 17 -24 | 6.69 | <0.001 | <0.001 |
| **Frontal** | 47 | Left inferior frontal gyrus | -29 18 -15 | 7.34 | <0.001 | <0.001 |

**Abbreviations**: SWA = slow wave activity; BA = Brodmann area; Coord. = coordinates; REM = rapid eye movement; NREM = non-rapid eye movement; Amg = amygdala

^v^ voxel-level; ^c^ cluster-level

***Comprehensive Clinical History for the patient:***

A 45-year-old female right-handed patient without history of seizures, traumatic brain injury, or central nervous infections was referred for the first time by her primary care physician to a neurology clinic in 2000 due to episodes of dizziness, light-headedness and flushes of warmth followed by chills, diaphoresis, and spells of “blank out” or “dissociation events” in which she felt she lost touch. Events lasted about two minutes and typically persisted over two days, perimenstrual. Episodes had been present for at least 5 years. MRI showed no relevant findings. EEG showed mild findings suggestive of a probable partial epilepsy associated to catamenial seizures. Lamotrigine (LGT) was prescribed, however, the symptoms remained unchanged and a novel feeling of fear appeared. The occurrence of her symptoms varied with regard to her menstrual cycle. In 2003, she also presented dysarthria. Episodes now occurred during sleep, and woke her up, causing symptomatic fatigue during the day. She was prescribed progesterone by her gynecologist as an aid for her menopause, with which she noticed the “spells” disappeared. LTG was tapered and a Zonisamide trial was attempted but proved unsuccessful.

In 2004, the above-described episodes subsided, replaced with dysarthria and a fine tongue tremor. This apparently coincided with a stressful period in the patient’s life. She was prescribed Carbamazepine (CBZ), unsuccessfully. Propranolol was started and the patient was referred to an Abnormal Movement Disorders Clinic, presenting with tongue and facial twitching predominantly on the right side. Propranolol was unsuccessful. An MRI was completed again. No significant findings were identified. The patient was diagnosed with hemifacial spasm and received Botox injections. However, an alternative physician noted the bilaterality of the condition and diagnosed lower facial myokymia. A voltage-gated K channel antibody syndrome was considered, and anti-GAD and VGK+ Channel antibodies were requested, but these tests were never completed.

In 2005, she was referred to Mayo Clinic, where a computer aided-subtraction ictal SPECT, co-registered to MRI, (SISCOM) was performed. This showed increased perfusion in the left inferior frontal lobe, thought to correspond with her facial twitching. An epilepsy diagnosis was confirmed, for which she was prescribed Levetiracetam (LEV). Due to worsening mood, it was discontinued. She was then prescribed CBZ once again. She was also unable to tolerate this medication and was changed to Topiramate and then finally to Phenytoin (PHE).

Nonetheless, her right facial seizures continued, unabated. In 2011, clonazepam was added to PHE for focal seizures lasting more than 90 seconds. A magnetic resonance angiography was completed and was unremarkable. A vertigo component became part of her seizure symptoms as well. Facial twitches continued into 2012, and falls occurred twice daily. Her PHE was increased. After this, facial seizures extended to the right sternocleidomastoid, and with this she noticed a sensation of her throat “closing”. Per report, seizures were triggered by stress as well as chewing food with her right side. PHE was stopped, LTG and vitamins were added, and she was referred to psychotherapy. The following year, seizures occurred almost every other day. A tightness in her mouth and neck, and a “pulling” that interfered with her talking presented; she also described dizziness, continued falls, and ongoing cognitive difficulties. LTG was increased, with little change in symptoms. In 2014, Oxcarbazepine (OXC) was added. It was ineffective.

An inpatient Epilepsy Monitoring Unit stay at the University of Wisconsin was arranged in 2015. During the EEG video monitoring procedure LTG was suspended. Buspirone, diphenhydramine, enoxaparin, and venlafaxine were continued as scheduled medications. Lidocaine and lorazepam were continued as needed. The EEG procedure showed seizures in which upon chewing, especially hard food and “the juicy taste of pineapple makes it worse”, she developed twitching of the right mouth and cheek. Events were 20-30 seconds each; the patient maintained awareness and responsiveness, though displayed expressive dysphagia. EEG seizure activity proved difficult to distinguish due to muscle artifact. However, left frontal and temporal spikes appeared during sleep and wake. An overnight HD-EEG recording was completed during this hospitalization. A Diagnosis of focal reflex epilepsy is made. LEV was restarted, as opposed to LTG. By 2016 her LEV dose was increased. Management for depression was undertaken with venlafaxine under the direction of a psychiatrist.

## In June 2016 events were reported to be “much better,” occurring 2-3 times weekly, at most, presenting with tingling and tightness in her jaw without jerking. When jerking did occur, it occurred in the setting of eating. Yet, by 2017, even with increasing LEV, her seizures worsened, occurring once daily. Increasing doses of OXC were tried as a replacement to LEV. She felt dizzy, fatigued, and experienced poor balance upon the medication change, so in 2018 she was put back on LEV. Seizures improved, however, she then moved to a different state and was lost to follow up.


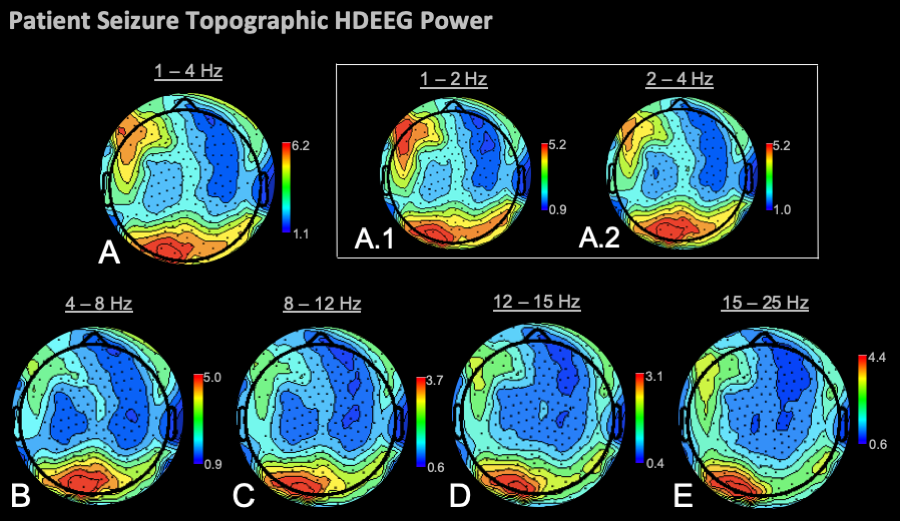


**Supplementary Figure 1. Topographical distribution of frequency bands during the patient’s seizure.** **(A)** Topographical distribution of patient SWA, in the isolated seizure state. **(A.1)** 1-2 Hz frequeny band, topographically; **(A.2)** topographic distribution of 2-4 Hz. **(B)** Theta band power during the patient’s seizure. **(C)** Alpha band power. **(D and E)** Low and high beta frequency power, respectively, distribution during the patient’s seizure.


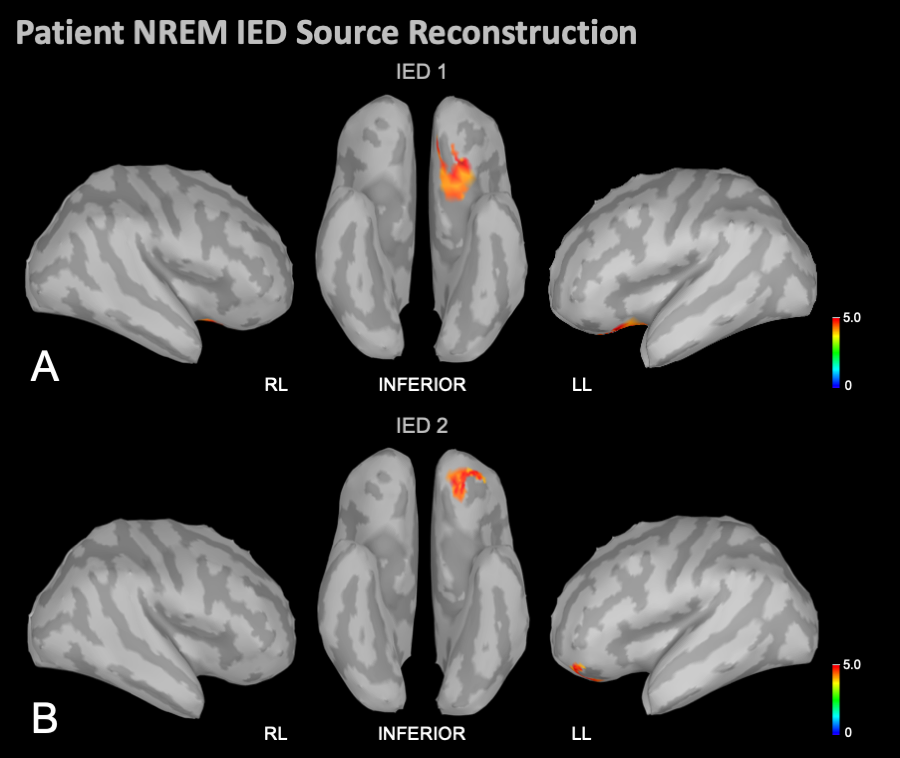


**Supplementary Figure 2. Source reconstruction of interictal epileptic discharges for the patient during NREM sleep.** Source reconstruction was completed identically to methodology described in the Methods section of this paper. Sources were computed at the maximum slope of the discharge, as described in previous work within our lab (1). **(A)** IED one, captured during NREM sleep, localized to the left inferomedial frontal lobe. **(B)** IED two, captured during NREM sleep, localized to the orbitofrontal area.

Abbreviations: IED = interictal epileptic discharge; RL = right lateral; LL = left lateral

1. Kang X, Boly M, Findlay G, Jones B, Gjini K, Maganti R, et al. Quantitative spatio-temporal characterization of epileptic spikes using high density EEG: Differences between NREM sleep and REM sleep. Sci Rep. 2020;10(1):1673.
